# CRIPTO-1 Is Immunolocalized in the Syncytiotrophoblast of Ampullary Pregnancies

**DOI:** 10.1155/2022/4769790

**Published:** 2022-04-08

**Authors:** Fábio Roberto Cabar, Pedro Paulo Pereira, Carla Letícia Bandeira, Estela Bevilacqua, Regina Schultz, Rossana Pulcineli Vieira Francisco

**Affiliations:** ^1^Discipline of Obstetrics, Department of Obstetrics and Gynecology, School of Medicine, University of São Paulo, São Paulo, SP, Brazil; ^2^Division of Obstetric Clinic, Hospital das Clínicas HCFMUSP, School of Medicine, University of São Paulo, São Paulo, SP, Brazil; ^3^Department of Cell and Developmental Biology, Institute of Biomedical Sciences, University of São Paulo, São Paulo, SP, Brazil; ^4^Division of Pathology, Hospital das Clínicas HCFMUSP, School of Medicine, University of São Paulo, São Paulo, SP, Brazil

## Abstract

**Introduction:**

Controlling the invasive activity of trophoblastic tissue has not been elucidated. In the accreta placenta, the invasion of placental tissue is directly related to the expression of CRIPTO-1 at the maternal-fetal interface. The aim of this study is to evaluate if the expression of the CRIPTO-1 is related to different degrees of trophoblast invasion into the tube wall in ampullary pregnancy.

**Methods:**

Prospective study with 21 patients with ampullary tubal pregnancy undergoing salpingectomy. Anatomopathological evaluation determined the degree of invasion of trophoblast tissues into the tubal wall and grouped the samples into invasive degrees I, II, or III. The groups were compared for tissue expression of CRIPTO-1 using the Kruskal-Wallis nonparametric test. *p* values lower than 0.05 were considered significant.

**Results:**

Quantitative expression of CRIPTO-1 differed in each of the three groups of trophoblast invasion in the tubal wall in ampullary pregnancies (*p* < 0.001). There is a difference between groups when grade I + grade II versus grade III (*p* < 0.001) and grade I versus grade II + grade III (*p* < 0.001). The tissue expression of CRIPTO-1 in ectopic trophoblasts showed that deeper invasion of the tubal wall was associated with stronger expression than in shallow invasion (*p* < 0.001). *Discussion*. In ampullary pregnancies, the depth of penetration of trophoblast tissue in the tubal wall is related to CRIPTO-1 tissue expression.

## 1. Introduction

Ectopic pregnancy (EP) results from implantation of the embryo in a location other than the endometrium, with the ampullary portion being the most common region (70%) [1]. Impairment of the function of the uterine tube depends on the degree of invasion of the trophoblast [2], whereby deeper invasion is associated with greater histological architecture and functional breakdown of the tube tissues.

Although the mechanisms controlling the invasive activity of trophoblastic tissue have not been completely elucidated, the loss of regulatory signals between the cells and decidual components has been proposed, as well as the participation of growth factors, hormones, cytokines, adhesion molecules, and oncogenes of the components of the maternal-fetal interface [3–6].

CRIPTO-1, a member of the epidermal growth factor family, is abundantly expressed in embryonic stem cells and tumor cells [7, 8]. In the accreta placenta, immunohistochemistry has revealed that invasion of placental tissue into the endometrium and myometrium is directly related to expression of CRIPTO-1 at the maternal-fetal interface [9]. In general, this expression is distributed among decidual cells and syncytiotrophoblasts and, primarily, invading extravillous cytotrophoblast (EVT) cells. The relationship between the degree of invasion and reactivity to CRIPTO-1 in EVT cells led the authors to suggest its participation in controlling the invasive properties of the trophoblast.

Therefore, in this study, we tested the hypothesis that the oncogenic factor CRIPTO-1 is related to different degrees of trophoblast invasion into the tube wall in ampullary pregnancy.

## 2. Methods

A prospective, cross-sectional, and comparative study was carried out involving patients diagnosed with ampullary pregnancies at the Obstetric Clinic of the Hospital das Clínicas of the Faculty of Medicine of the University of São Paulo (HCFMUSP) from July 2012 to August 2013. The study was performed after approval by the Ethics Committee for Analysis of Research Projects at HCFMUSP (CAPPesq) under number 51757/12.

In this study, we examined trophoblast invasion exclusively in ampullary pregnancies. The inclusion criteria were spontaneously conceived pregnancies (excluded assisted reproduction techniques) because the use of ovulation-inducing drugs stimulates the supraphysiological production of steroid hormones, and the influence of these hormones on the production and expression of CRIPTO-1 is unknown; radical surgical treatment (salpingectomy), with a diagnosis of EP in the ampulla region confirmed during the surgery. Patients with any chronic, immune, or infectious diseases either associated or not with the use of continued medicines were excluded from the study.

Patients treated with suspected EP were assessed using the algorithm recommended for the diagnosis of EP [10]. After confirming the diagnosis, the patients were referred for conservative (clinical or surgical) or radical treatment according to their hemodynamic status, desire to preserve reproductive potential, level and evolution of serum *β*-hCG, amount of free fluid in the pelvis, presence of a fetal heartbeat, and ectopic mass size assessed by ultrasound.

During the study period, 60 women were diagnosed with EP and 42 were eligible for the study and of these 18 patients who were not preselected, 15 were clinically treated and the other three were treated with methotrexate (MTX). Of the 42 patients who were eligible, the first seven for each histological group were included in chronological order, comprising 21 patients.

The degree of invasion of trophoblast was determined with the anatomopathological classification, and was grouped into invasive degrees I, II, or III.

After salpingectomy, the tissues were sent in 10% formalin solution for anatomopathological classification. Examination of the uterine tubes affected consisted of i) macroscopic analysis with determination of the affected area, maximum dilation area, and measurements of the fallopian tube and ii) microscopic analysis of samples routinely processed for paraffin embedding and hematoxylin-eosin (HE) staining to confirm the degree of tube wall invasion. On average, ten sections of each uterine tube were analyzed. To facilitate the identification of the tissue invaded by the trophoblast, the histological material was also stained using the Masson technique to identify the muscle fibers of the fallopian tubes. In addition, immunohistochemical staining for human placental lactogen (hPL) was performed to enable the identification of intermediate trophoblasts and to determine the depth of invasion of trophoblastic tissue.

The analysis was performed by a single and experienced medical pathologist who had no information about the patients. The samples were classified according to the depth of trophoblast invasion into the wall of the uterine tube, adopting the following criteria [11]: grade I, trophoblast infiltration limited to the mucosa; grade II, trophoblast infiltration up to the muscular layer; or grade III, trophoblast infiltration involving the entire thickness of the uterine tube, with or without rupture of the serosa.

After classification, the slides were randomized and received code numbers to be analyzed without any identification.

For immunohistochemical reactions, 5-*μ*m-thick sections were collected on silanized histological slides and deparaffinized, hydrated, and subjected to immunohistochemistry to localize CRIPTO-1 (ABCAM) and cytokeratin-7 (Dako). Additional slides were stained with hematoxylin and eosin (Sigma) to examine the morphology of the materials. The procedures are briefly described in Table 1.

The samples were analyzed using a conventional light microscope Axioshop 2 (Carl Zeiss) coupled to the capture system Axio Vision 4.7 (Carl Zeiss). Images were acquired with a 10x magnification lens, with 1388 × 1040 pixels and a resolution of four pixels/*μ*m^2^. Quantification was performed using five images for each sample. Quantitative analysis was carried out using ImageJ 1.43 software (NIMH, NHI, Bethesda, MD, USA, http://rsweb.nih.gov/ij/). The immunoreactive areas were segmented into different channels using the Color Deconvolution plug-in (Gabriel Landini, Http://http://www.dentistry.bham.ac.uk/landing/software/software), thus estimating the number of pixels contained within the segmented perimeter, which were normalized by the total tissue area; the results are expressed in square micrometers (*μ*m^2^). Identification of the samples with regard to the degree of invasiveness was only performed at the time of statistical analysis.

Quantitative variables are summarized as means, medians, standard deviations, and minimum and maximum values. Qualitative variables are presented as absolute frequencies (*n*) and percentages (%). The three groups were compared for tissue expression of CRIPTO-1 using the Kruskal-Wallis nonparametric test. The Mann–Whitney nonparametric test was employed to assess equality between groups, two by two. The level of significance adopted was 5%, such that *p* values less than 0.05 were considered significant. The data analysis was performed using IBM SPSS software version 20.

## 3. Results

**Figure 1:**
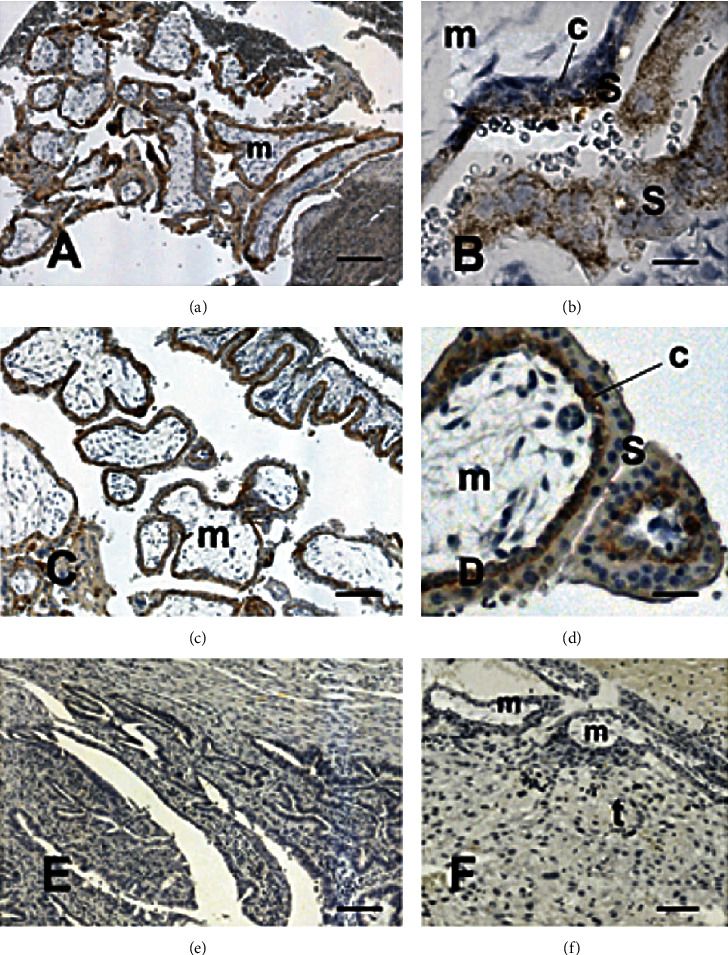
Expression of CRIPTO-1 ((a), (b), and (e)) and cytokeratin ((c) and (d)) in placental samples of the tubal wall of ampullary pregnancies. Localization of these markers was identified based on brownish staining. CRIPTO-1 staining ((a) and (b)) indicates its expression only in syncytiotrophoblasts (S). No CRIPTO-1 was detected on the fallopian tube ((e), t), in the villous mesenchymal cells (m) and in the cytotrophoblast (c). (f) Negative control of the immune reaction, in which the primary antibody was omitted. Scale bar, 350 mm ((a), (c), and (e)), 30 mm (b), 40 mm (d), and 150 mm (f).

**Figure 2:**
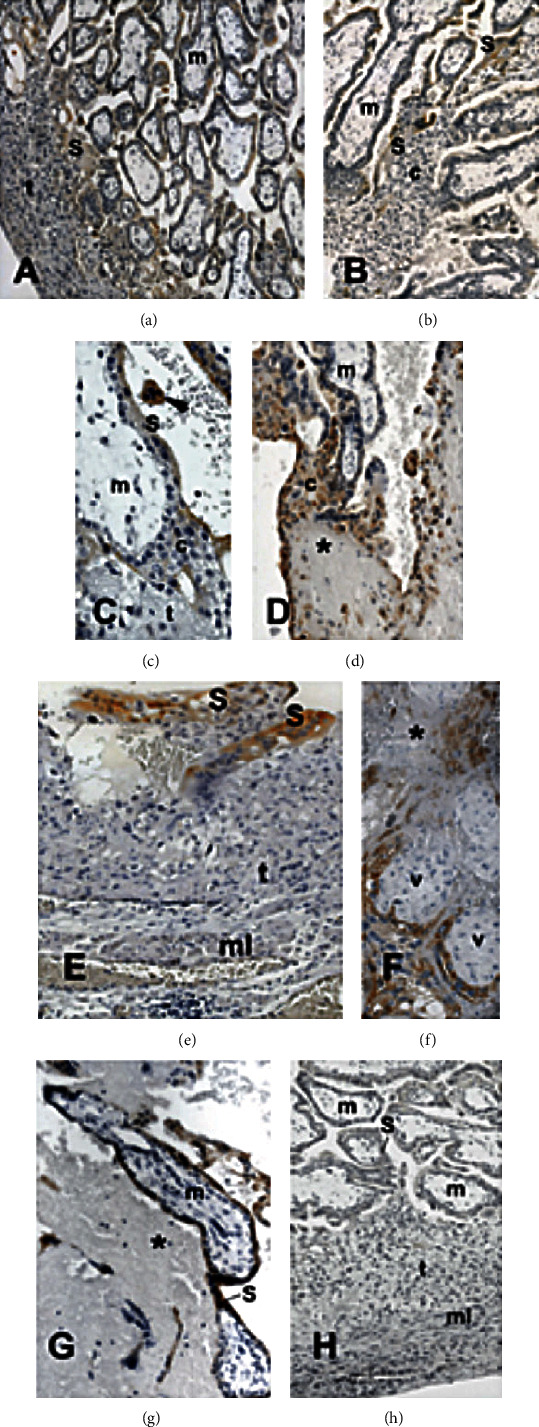
Expression of CRIPTO-1 ((a)–(c) and (e)–(h)) and cytokeratin (d) in placental samples of the tubal wall of ampullary pregnancies. Localization of these markers was identified based on brownish staining. The chorionic villi establish different interactions with the tube tissues ((a), (c), (e), (f), and (h)) or with areas in degeneration (∗d, g). At the apical pole of the villi ((b)–(d)), the cytotrophoblast (c) grows similarly to what is seen in uterine pregnancy. Cytokeratin staining (d) highlights the accumulation of migratory cytotrophoblast cells on the tubal tissue. (h) Negative control of the immune reaction, in which the primary antibody was omitted. (t) Fallopian tube, (m) villous mesenchymal cells, (S) syncytiotrophoblast, (arrowhead) syncytial knot. Scale bar, 250 mm ((a), (b), (d), and (h)), 75 mm ((c), (e), and (f)), and 100 mm (g).

Immunohistochemical analysis revealed the presence of the CRIPTO-1 protein exclusively in fetal tissues (Figures 1(a) and (b) and 2(e)). Indeed, no reaction was observed in the cells of the villous or extravillous cytotrophoblast, as identified by cytokeratin 7, in semiserial sections (Figures 1(c) and 1(d)).

The chorionic villi established contact with the uterine tube tissues with apparently healthy histological organization (Figures 2(a), 2(e), and 2(h)); tubal tissue with extensive areas of necrosis or with hemorrhagic areas was observed (Figures 2(d), 2(f), and 2(g)). In all conditions, CRIPTO-1 reactivity was seen either in the syncytiotrophoblast layer or in syncytial knots in the intervillous space (Figures 2(a)–(c) and 2(e)–(g)). The growth of the extravillous cytotrophoblast at the end of the chorionic villi (Figures 2(b) and 2(c)) was detected in cytokeratin 7 immunoreactions (Figure 2(d)). This reaction also highlighted the formation of a cytotrophoblast layer in the tubal tissue in contact with the chorionic villi, similar to the trophoblastic shell observed in uterine implantation.

**Figure 3:**
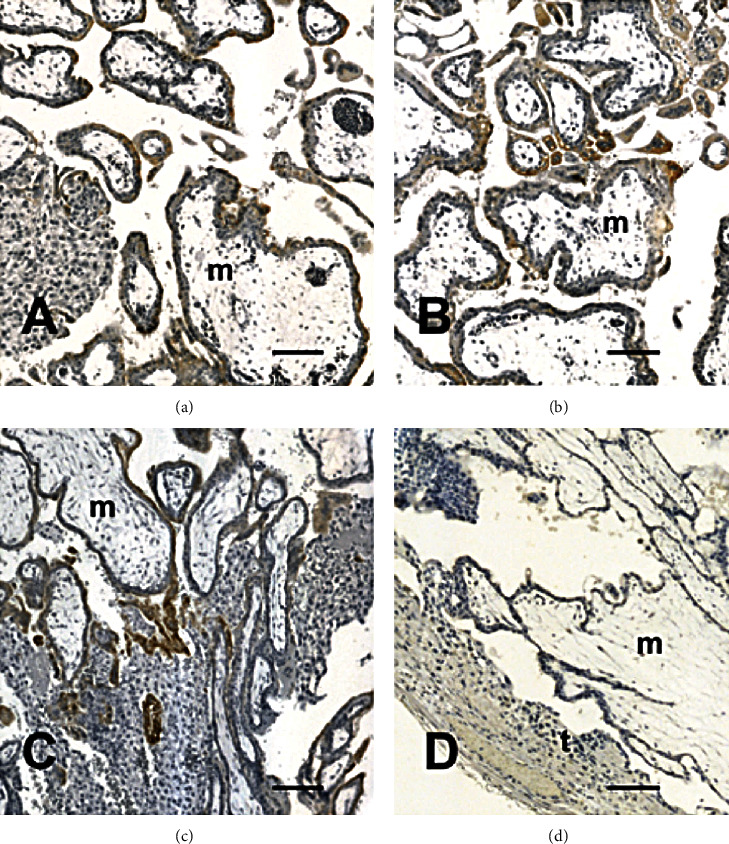
Expression of CRIPTO-1 in placental samples of the tubal wall of ampullary pregnancies. Localization of these markers was identified based on brownish staining. The staining was not homogeneous all throughout the villous surface ((a) and (b)). Note that in (c), CRIPTO-1 staining is weak on the surface areas of some villi, but strong at the interface with the tube mucosa. (d) Negative control of the immune reaction, in which the primary antibody was omitted. (t) Fallopian tube, (m) villous mesenchymal cells. Scale bar, 250 mm ((a)–(d)).

Immunoreactivity occurred at different degrees of intensity and displayed a broad distribution in the syncytial layer, covering free chorionic villi and in contact with the uterine mucosa or blood clots. Some specimens were weakly reactive for CRIPTO-1, whereas the reaction was intense in others, varying from some reactive chorionic villi to essentially all staining positively (Figure 3).

Control reactions in which the primary antibody was omitted did not show any reactivity (Figures 1(f), 2(h), and 3(d)).

Quantification in pixels of this reactivity ranged from 0.22 to 6.01 pixels/*μ*m^2^ (average 2.91 pixels/*μ*m^2^). When reactivity was related to the degree of trophoblast invasion determined by pathological analyses, grade I showed values that were significantly lower than those of grades II and III.  Table 2 shows the quantitative tissue expression of CRIPTO-1 according to the degree of invasion of trophoblasts in the tubal wall affected by an EP.

According to the Kruskal-Wallis test, quantitative expression of CRIPTO-1 differed in each of the three groups of trophoblast invasion in the tubal wall in ampullary EPs (*p* < 0.001). Subsequently, the Mann–Whitney test demonstrated a difference between groups when grade I + grade II versus grade III (*p* < 0.001) and grade I versus grade II + grade III (*p* < 0.001) were compared (Tables 3 and 4).

## 4. Discussion

In this study, we found no evidence that the invading cytotrophoblast cell population expresses CRIPTO-1 in ampullary fallopian tube EPs, as found in uterine pregnancy and accreta placentas [9]. However, CRIPTO-1 expression was observed in the syncytiotrophoblast layer, apparently maintaining a close relationship with the degree of invasion of the tube wall. These findings strongly suggest that different mechanisms and interactions occur in the maternal-fetal relationship in EP and that CRIPTO-1 may be involved in the dysfunctional invasion occurring in these pregnancies.

Studies carried out to date have not found support to explain the modulation of trophoblast invasion in the wall of the uterine tube. As occurs in uterine implantation sites, differences between the fallopian tube regions with and without the implanted embryo were only found in the tissue concentration of VEGF and VEGF gene expression and that of its receptors KDL and Flt-1 [12]. Similarly, expression of matrix metalloproteinases (MMP-2, MMP-9, and MMP-14), which facilitate migration by breaking down barriers formed by the extracellular matrix, or the ratio with their inhibitors (TIMP-1, TIMP-2, and TIMP- 3), did not show significant differences with regard to the degree of tubal invasion [13].

CRIPTO-1 expression is usually low or absent in healthy tissues; however, it is secreted at high levels by the colon, ovary between other tumors [14–16]. At the human maternal-fetal interface, CRIPTO-1 has already been reported to immunolocalize in the syncytial layer, mainly in extravillous cytotrophoblast cells from healthy pregnancies and in pregnancies with alterations in invasiveness [9]. As extravillous cytotrophoblast cells are highly invasive in early gestation, the authors of one study speculated whether this expression is due to the similarities between the trophoblast invasive process and tumor cells [17].

In EP, however, we detected expression of CRIPTO-1 only in the syncytiotrophoblast layer, for which some other considerations should be examined. Loss of cytotrophoblast invasiveness cannot be a factor because these cells were immunolocalized by cytokeratin staining in the tubal mucosa; in several cases, a trophoblast shell-like layer formed, as seen in healthy uterine pregnancies. Several factors might be involved in the downregulation of CRIPTO-1 in these cells, likely related to the specific tubal microenvironment. Accumulating evidence shows that the profile of genes and proteins in the tubal and uterine mucosa lodging an embryo may be territory specific, leading to threshold values at which gene expression may be impaired [18, 19]. In addition, other specific territorial characteristics, such as uterine NK cells (which exhibit a highly specific repertoire of immune activities, Sharma et al. [20]), endothelial cells (which can express territory-dependent profiles of inflammatory and regulatory proteins, Zhang et al. [21]), and uterine gland cells (which express a large number of growth factors and regulatory molecules, Filant and Spencer [22]), may be considered. Another relevant fact is how gene and protein pathways of the fallopian tube may be regulated by products from the ectopic placenta, which may vary from the uterine response in healthy pregnancies. Nevertheless, which of them has a regulatory action on CRIPTO-1 expression by extravillous cytotrophoblasts in the tubal mucosa is an issue that needs further investigation.

Expression of CRIPTO-1 in villous syncytiotrophoblasts is observed in the early human placenta [9]. However, its participation in endometrial invasion is considered limited to the first stages of implantation due to the embryo's intrusion and anchorage in the decidua. Undoubtedly, its primary function from this stage onward is to coat the intervillous space and facilitate molecular exchange, protection, and hormonal production during pregnancy [23].

Nevertheless, we found clear differential expression of CRIPTO-1 in the syncytial layer related to the degree of invasion/injury to the fallopian tube. Cripto-1 reactivity in different intensities and distributions in the placental tissue was found, regardless of the relationships with either a healthy mucosa, areas with inflammatory infiltration, necrotic tissues, and blood clots. However, no morphological evidence of invasive activity can be inferred from our analyses.

An open question is why CRIPTO-1 expression might be related to the degree of invasion/injury of the fallopian tube. One possibility is that the degree of injury, signaled by different ways in the microenvironment (variations in tissue oxygenation, inflammatory and immune mediators, etc.). Multiple pathways control expression of CR-1 [24]. Among them, it has been shown that HIF-1*α* plays an essential role in activating CR-1 expression and is activated (stabilized) when the O_2_ concentration declines under inflammatory conditions [25]. In summary, expression of CRIPTO-1 appears to be more related to the injury status of the tube caused by cytotrophoblast invasion than by invasion per se. Nevertheless, these results suggest different mechanisms of CRIPTO-1 regulation in villous and extravillous trophoblast cells, a hypothesis that remains to be elucidated.

This study adds information about the behavior of trophoblasts in EP. It also opens up another important issue: the possibility of CRIPTO-1, produced by the syncytiotrophoblast, being secreted into the bloodstream of pregnant women in different patterns and whether this pattern is similar to that found in healthy pregnancies.

The information is in perfect compliance with that found by Bandeira et al. [9] in the analysis of topical pregnancies (with placental accretion). The present study was not able to verify whether all the pathophysiological and histopathological mechanisms occur in the same way as those observed in the 2014 study, as this issue was outside the focus of the original objective.

This investigation, despite having been carried out with a limited group of women, presents encouraging results, with a connection to the hypothesis that was initially posited. However, this investigation allowed us to identify only a small piece of this huge puzzle, that is, the invasion of trophoblastic tissue in the tubal wall. Undoubtedly, further studies will be necessary to better understand the mechanisms of synergism and antagonism in the invasion of trophoblasts in the uterine tube in EP.

## Figures and Tables

**Table 1:** Description of Immunolocalization procedures.

Procedures	Description
1	Rescue of antigenic sites in pH 6.0 citrate buffer (Biogen) and Trilogy reagent (Cell Marque) in an electric pressure cooker (high pressure) for 15 minutes;
2	Endogenous peroxidase block using 10% hydrogen peroxide (H_2_O_2_, Sigma) diluted in phosphate-buffered saline (PBS, Sigma) 0.1 M (v/v) for 10 minutes at room temperature
3	Blocking of nonspecific antigenic sites by incubation with 2% bovine serum album in (BSA, Sigma) in PBS (m/v) for one hour at room temperature, in a humid chamber
4	Incubation with the primary rabbit monoclonal anti-human CRIPTO-1 antibody (ABCAM) at a concentration of 1 : 100 in PBS overnight at 4°C in a humid chamber or with the primary mouse monoclonal anti-human cytokeratin antibody (Dako) at a concentration of 1 : 350 in PBS for one hour at room temperature in a humid chamber;
5	Incubation with the REVEAL- Biotin-Free Polyvalent DAB (Spring) kit and subsequent development of peroxidase activity with 3,3′-diaminobenzidine using the Sigma Fast TM reagent (Sigma), both according to the manufacturer's recommendations;
6	Counterstaining with Mayer's hematoxylin (Sigma) and mounting with glass coverslips and Entellan® (Merck).

**Table 2:** CRIPTO-1 tissue expression and degree of infiltration of the trophoblast (*n* = 21). HCFMUSP - July 2012 to August 2013.

Trophoblast invasion degree	CRIPTO-1 [pixels/*μ*m^2^]	
	Mean ± S.D.	Median ^a^
I	1.22 (0.64)	1.17 (0.22–2.16)
II	2.79 (0.79)	2.86 (1.44–3.95)
III	4.73 (0.65)	4.57 (3.99–6.01)

^a^(minimum and maximal values); S.D.: standard deviation. Kruskall-Wallis test; *p* < 0.001.

**Table 3:** CRIPTO-1 tissue expression and degree of trophoblast tissue infiltration (*n* = 21). GI + GII versus GIII. HCFMUSP - July 2012 to August 2013.

Trophoblast invasion degree	CRIPTO-1 [pixels/*μ*m^2^]	
	Mean ± S.D.	Median ^a^
I + II	2.00 (1.07)	1.98 (0.22–3.95)
III	4.73 (0.65)	4.57 (3.99–6.01)

^a^(minimum and maximal values); S.D.: standard deviation. Mann–Whitney test, *p* < 0.001.

**Table 4:** CRIPTO-1 tissue expression and degree of trophoblast tissue infiltration (*n* = 21). GI versus GII + GIII. HCFMUSP - July 2012 to August 2013.

Trophoblast invasion degree	CRIPTO-1 [pixels/*μ*m^2^]	
	Mean ± S.D.	Median ^a^
I	1.22 (0.64)	1.17 (0.22–2.16)
II + III	3.76 1.22)	3.97 (1.44–6.01)

^a^(minimum and maximal values); S.D.: standard deviation, Mann–Whitney test, *p* < 0.001.

## Data Availability

The access to data is restricted because of legal and ethical concerns, specially involving patient privacy. Brazilian Civil and Criminal Law, Medical Code of Ethics prohibits the divulgation of patient information.

## References

[1] Cunningham F., Leveno K. J., Bloom S. L., Cunningham F. (2018). Ectopic pregnancy. *Williams Obstetrics*.

[2] Green L. K., Kott M. L. (1989). Histopathologic findings in ectopic tubal pregnancy. *International Journal of Gynecological Pathology*.

[3] Ohlsson R. (1989). Growth factors, protooncogenes and human placental development. *Cell Differentiation and Development*.

[4] Mühlhauser J., Crescimanno C., Kaufmann P., Höfler H., Zaccheo D., Castellucci M. (1993). Differentiation and proliferation patterns in human trophoblast revealed by c-erbB-2 oncogene product and EGF-R. *The Journal of Histochemistry and Cytochemistry*.

[5] Marzioni D., Capparuccia L., Todros T., Giovannelli A., Castellucci M. (2005). Growth factors and their receptors: fundamental molecules for human placental development. *Italian Journal of Anatomy and Embryology*.

[6] Tseng J. J., Chou M. M., Hsieh Y. T., Wen M. C., Ho E. S., Hsu S. L. (2006). Differential expression of vascular endothelial growth factor, placenta growth factor and their receptors in placentae from pregnancies complicated by placenta accreta. *Placenta*.

[7] Watanabe K., Meyer M. J., Strizzi L. (2010). Cripto-1 is a cell surface marker for a tumorigenic, undifferentiated subpopulation in human embryonal carcinoma cells. *Stem Cells*.

[8] Rangel M. C., Karasawa H., Castro N. P., Nagaoka T., Salomon D. S., Bianco C. (2012). Role of Cripto-1 during epithelial-to-mesenchymal transition in development and cancer. *The American Journal of Pathology*.

[9] Bandeira C. L., Urban Borbely A., Pulcineli Vieira Francisco R., Schultz R., Zugaib M., Bevilacqua E. (2014). Tumorigenic factor CRIPTO-1 is immunolocalized in extravillous cytotrophoblast in placenta creta. *BioMed Research International*.

[10] Pereira P., Zugaib M., Bittar R. (2013). Gravidez ectópica. *Protocolos assistenciais da clínica obstétrica da faculdade de medicina da USP*.

[11] Natale A., Candiani M., Merlo D., Izzo S., Gruft L., Busacca M. (2003). Human chorionic gonadotropin level as a predictor of trophoblastic infiltration into the tubal wall in ectopic pregnancy: a blinded study. *Fertility and Sterility*.

[12] Lam P. M., Briton-Jones C., Cheung C. K., Leung S. W., Cheung L. P., Haines C. (2004). Increased messenger RNA expression of vascular endothelial growth factor and its receptors in the implantation site of the human oviduct with ectopic gestation. *Fertility and Sterility*.

[13] Gomez U. (2019). *Avaliação da expressão das metaloproteinases e de seus inibidores na interface maternoembrionária em gestações ectópicas ampulares*.

[14] Saloman D. S., Bianco C., Ebert A. D. (2000). The EGF-CFC family: novel epidermal growth factor-related proteins in development and cancer. *Endocrine-Related Cancer*.

[15] Normanno N., De Luca A., Bianco C. (2004). Cripto-1 overexpression leads to enhanced invasiveness and resistance to anoikis in human MCF-7 breast cancer cells. *Journal of Cellular Physiology*.

[16] Mallikarjuna K., Vaijayanthi P., Krishnakumar S. (2007). Cripto-1 expression in uveal melanoma: an immunohistochemical study. *Experimental Eye Research*.

[17] Bischof P., Campana A. (2005). A putative role for oncogenes in trophoblast invasion?. *Hum Reprod*.

[18] Salih S. M., Taylor H. S. (2004). HOXA10 gene expression in human fallopian tube and ectopic pregnancy. *American Journal of Obstetrics and Gynecology*.

[19] Savaris R. F., Hamilton A. E., Lessey B. A., Giudice L. C. (2008). Endometrial gene expression in early pregnancy: lessons from human ectopic pregnancy. *Reproductive Sciences*.

[20] Sharma S., Godbole G., Modi D. (2016). Decidual control of trophoblast invasion. *American Journal of Reproductive Immunology*.

[21] Zhang J., Burridge K. A., Friedman M. H. (2008). In vivo differences between endothelial transcriptional profiles of coronary and iliac arteries revealed by microarray analysis. *American Journal of Physiology. Heart and Circulatory Physiology*.

[22] Filant J., Spencer T. E. (2014). Uterine glands: biological roles in conceptus implantation, uterine receptivity and decidualization. *The International Journal of Developmental Biology*.

[23] Frank H.-G., Polin R. A., Abman S. H., Rowitch D. H., Benitz W. E., William W. (2017). Placental development. *Fetal and Neonatal Physiology*.

[24] Loying P., Manhas J., Sen S., Bose B. (2015). Autoregulation and heterogeneity in expression of human Cripto-1. *PLoS One*.

[25] Triner D., Shah Y. M. (2016). Hypoxia-inducible factors: a central link between inflammation and cancer. *The Journal of Clinical Investigation*.

